# Conditions of Service of Army Compounders

**Published:** 1914-10-03

**Authors:** 


### INSTITUTIONAL PHARMACISTS AND THE WAR.

### Conditions of Service of Army Compounders.

The experience in rapid manipulation which hospital dispensers have had the opportunity of obtaining in the daily routine of institutional work is especially valuable in times of crisis, and such institutional pharmacists as have joined one or other of the branches of service will no doubt do credit to their calling. That the War Office is in need of dispensers was made clear by the appeal that was issued on the outbreak of hostilities, when this class was included in the list of craftsmen who were invited to enlist. Under the circumstances it will be of interest to set forth some particulars of the conditions of service of Army compounders. Efforts have been made on more than one occasion by the Pharmaceutical Society to secure for duly qualified pharmacists serving as military dispensers the rank of commissioned officer, but without success; and although there are strong reasons to believe that before long a renewal of similar representations may meet with a more favourable reception, Army dispensers at the present time are not enjoying the privilege to which their training and education seem to entitle them. But at a time when all the horrors of war are with us, and men are urgently needed to fill the ranks of those who are falling day by day, it would be churlish, if not unpatriotic, to put a price on our services which could not be paid in times of peace. In the Regular Army dispensers are non-commissioned officers in the Royal Army Medical Corps. They enlist as ordinary soldiers but are required to pass certain tests, not of a very searching character, in general intelligence and to obtain certificates of suitability as members of the corps. Prior to undergoing training as dispensers the soldiers must have passed the examination for promotion for corporal in the Royal Army Medical Corps. There is also a period of nine months to be spent in dispensing in a military hospital, and this training includes other hospital and nursing duties. There are in all about a thousand, rather less than more, Army compounders, and most of them hold the rank of sergeant and upwards; the Army compounders' examination, it may be added, is admittedly inferior to that for the pharmaceutical diploma. What has been written above applies, of course, to the standing Army, but at the commencement of the war a special Army Order was issued in which the conditions of service of civilians enlisting for temporary service during the war were set forth.

Men enlisted under this Army Order must not be more than forty years of age, and enlistments are for one year or for the duration of the war; if the war is over in less than a year, the men may be discharged at once. Pharmacists desirous of appointment to the Royal Army Medical Corps, who are qualified as dispensers, are required to produce the Minor Certificate of the Pharmaceutical Society. They must enlist as ordinary pharmacists without conditions, but they should intimate in what capacity they desire to serve. When this Order was issued the services of 150 dispensers were stated to be required, not more than fifty of these being expected to serve with any expeditionary force sent out of the country. It is clear from the Order, however, that when enlisted they must be prepared to undertake any duties that may be required of them. Free rations of bread, meat, and groceries, at home and abroad, will be given as well as accommodation. Married men (and widowers) enlisting will have separation allowance. Clothing and necessaries will be issued free and a bounty of £5 will be paid on final approval, and pay (as dispensers) will be issued at the rate of 6s. a day. On discharge, except for misconduct, each man will receive a gratuity of £5, in addition to any war gratuity given to the troops at the end of the war. Men will be entitled to a pension if discharged in consequence of wounds, injuries, or disability received or contracted while on military duty, and if they die through war service or injury on duty their families will be pensioned. In the Territorial Force dispensers are also without commissioned rank. The British Red Cross Society again has room for the qualified dispenser. As stated in the Training Manual, issued with the approval of the War Office, the field ambulances of the Territorial Force are sufficient to meet the requirements of the troops on the march and in action, but they have no men to spare to remain behind to attend to the sick and injured along the line of communications.

It is with the intention of meeting this gap in the Territorial Force that the Voluntary Aid Detachments, in connection with the Red Cross movement, were formed. At the beginning of the present year there were nineteen hundred Voluntary Aid Detachments, of which over six hundred were men's detachments, each with a pharmacist attached to it. The duties of the pharmacist are stated in the official form as follows:—(*a*) In the temporary absence of the senior officers, their duties will devolve upon the pharmacist; (*b*) the pharmacist would dispense the medicines which may be ordered for the sick, keep charge of the instruments, appliances, dressings, etc.; (*c*) he will be responsible to the medical officer for their good order and readiness for use; (*d*) he will issue requisitions for any fresh supplies required, obtaining the sanction and signature of the medical officer to each requisition before issue; (*e*) he will attend the drills with the rest of the detachment, and when not dispensing shall carry out any other duties as arranged by the Commandant. Voluntary Aid Detachments, as far as possible, will be employed near their own homes, and while so employed would not receive payment or allowances; but where services rendered in war have caused loss of wages, reasonable claims which were endorsed by the Commandant would be admitted by the Army Council.

